# Current landscape of gene‐editing technology in biomedicine: Applications, advantages, challenges, and perspectives

**DOI:** 10.1002/mco2.155

**Published:** 2022-07-14

**Authors:** Weilin Zhou, Jinrong Yang, Yalan Zhang, Xiaoyi Hu, Wei Wang

**Affiliations:** ^1^ Department of Biotherapyy State Key Laboratory of Biotherapy and Cancer Center West China Hospital Sichuan University Chengdu People's Republic of China; ^2^ Department of Hematology Hematology Research Laboratory State Key Laboratory of Biotherapy and Cancer Center West China Hospital Sichuan University Chengdu Sichuan P. R. China; ^3^ Department of Gynecology and Obstetrics Development and Related Disease of Women and Children Key Laboratory of Sichuan Province Key Laboratory of Birth Defects and Related Diseases of Women and Children Ministry of Education West China Second Hospital Sichuan University Chengdu P. R. China

**Keywords:** clinical trials, disease diagnosis, disease modeling, gene therapy, genome editing, immunotherapy

## Abstract

The expanding genome editing toolbox has revolutionized life science research ranging from the bench to the bedside. These “molecular scissors” have offered us unprecedented abilities to manipulate nucleic acid sequences precisely in living cells from diverse species. Continued advances in genome editing exponentially broaden our knowledge of human genetics, epigenetics, molecular biology, and pathology. Currently, gene editing‐mediated therapies have led to impressive responses in patients with hematological diseases, including sickle cell disease and thalassemia. With the discovery of more efficient, precise and sophisticated gene‐editing tools, more therapeutic gene‐editing approaches will enter the clinic to treat various diseases, such as acquired immunodeficiency sydrome (AIDS), hematologic malignancies, and even severe acute respiratory syndrome coronavirus 2 (SARS‐CoV‐2) infection. These initial successes have spurred the further innovation and development of gene‐editing technology. In this review, we will introduce the architecture and mechanism of the current gene‐editing tools, including clustered regularly interspaced short palindromic repeats (CRISPR) and CRISPR‐associated nuclease‐based tools and other protein‐based DNA targeting systems, and we summarize the meaningful applications of diverse technologies in preclinical studies, focusing on the establishment of disease models and diagnostic techniques. Finally, we provide a comprehensive overview of clinical information using gene‐editing therapeutics for treating various human diseases and emphasize the opportunities and challenges.

## Graphical abstract

The expanding gene‐editing toolbox has driven a biomedical revolution. These molecular tools tremendously contribute to the progression of fundamental research, such as epigenetics, drug development, disease modeling, and disease diagnosis. Furthermore, therapeutic gene editing offers a profound opportunity to improve the current treatment of human diseases.

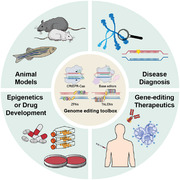

## 1 INTRODUCTION

An enormous amount of genetic information is hidden in the nucleotide sequence; sometimes, only a single base mutation has the potential to cause incurable diseases and even death. Over the past decade, the rapid expansion of gene‐editing technology has constantly reshaped our conception of human genetics. These exquisite molecular tools have driven a massive revolution in many industries, especially the therapeutic industry. The “genomic scalpel” offers a profound opportunity to investigate genetic information, expanding our understanding of gene functions. Continued efforts to understand and elucidate these disease‐causing mutations will be crucial to improving health care for patients with genetic disorders. Previous preclinical studies have demonstrated that the pathological process can be reversed using gene‐editing molecules to inactivate or correct morbid mutations. These promising results inspired the efforts to develop gene‐editing therapeutics to treat human genetic diseases in the clinic. In addition to genetic disorders, the scope of gene‐editing applications has been extended to other conditions, including cancer and viral infections. Scientists in both biomedical research and commercial exploitation have displayed great enthusiasm for therapeutic gene editing. However, to reach the ultimate goal in which all genetic disorders can be cured by personalized gene editing, many difficult problems still need to be addressed. For instance, clinical applications require safer and more accurate engineered enzymes and more efficient delivery approaches. Regulating this technology to avoid abuse is also a focus of public concern.

In this review, we outline the development of gene‐editing technology and introduce the most commonly used gene‐editing tools. Next, we describe the current state of genome editing applications in fundamental research, focusing on developing disease models and diagnostic techniques. Finally, we summarize the clinical application of therapeutic gene editing and highlight the opportunities and challenges.

## 2 CONSTANTLY UPDATED GENOME‐EDITING TECHNOLOGY

**FIGURE 1:**
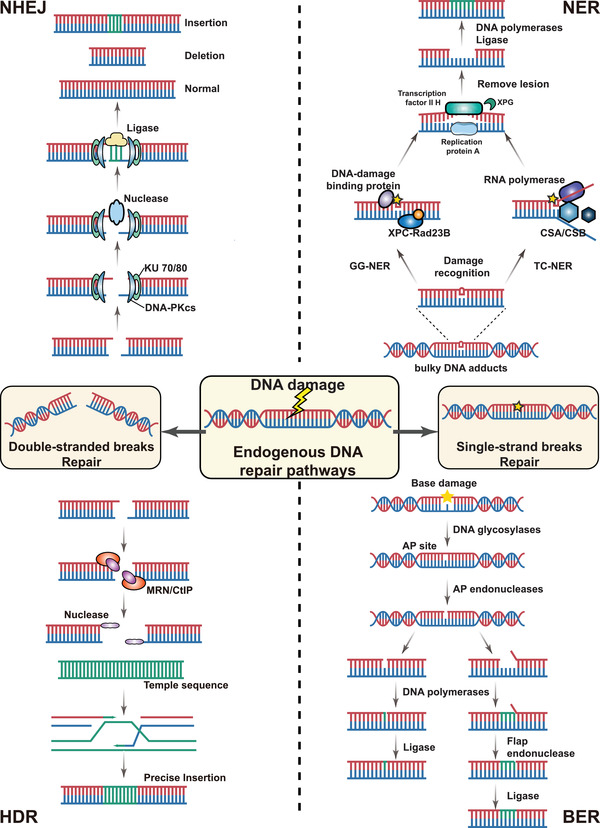
Overview of DNA damage repair mechanisms. DNA damage repair systems can be classified into two main groups: DNA double‐strand break (DSB) repair and single‐strand break (SSB) repair. Generally, DSBs are repaired by nonhomologous end‐joining (NHEJ) or homology‐directed repair (HDR). SSBs are repaired by nucleotide excision repair (NER) or base excision repair (BER). In the NHEJ pathway, DNA damage can be recognized by the Ku70/Ku80 complex and repaired by subsequent nucleases and ligases. The HDR pathway can achieve effective lesion repair using template‐directed DNA. The NER contains two signaling pathways, global genomic NER (GG‐NER) and transcription‐coupled NER (TC‐NER). GG‐NER used the heterotrimeric lesion recognition factor (consisting of XPC, RAD23, and CETN2) to detect DNA lesions, but TC‐NER used RNA polymerase II (RNAPII), Cockayne syndrome A (CSA) and CSB protein. The BER pathway is responsible for resolving nonbulky single‐base lesions. Gene editing agents use these pathways to achieve genetic modification

In the 1980s, restriction endonuclease from bacteria was used to cleave the DNA double helix.^1^ Since then, scientists have made a great effort to enrich the genome‐editing arsenal to manipulate gene sequences at specific loci. Different classes of genome manipulation tools are currently exploited for multiple applications in laboratory investigation, agriculture, and medicine. Common tools include zinc finger nucleases (ZFNs), transcription activator‐like (TAL) effector nucleases (TALENs), clustered regularly interspaced short palindromic repeat (CRISPR) and CRISPR‐associated (Cas) protein, base editors, and primer editors.^2^ It is clear that every gene‐editing method has different mechanisms and operational constraints.^3^ However, most genetic modifications from various tools (when not extended to base and primer editors) rely on generating a double‐strand break (DSB), which will trigger the endogenous repair mechanism in eukaryotic cells.^4^, ^5^ Two endogenous DNA repair pathways within mammalian cells are generally responsible for repairing DSBs, including nonhomologous end‐joining (NHEJ)‐mediated repair^6^ and homology‐directed repair (HDR; Figure 1).^7^, ^8^ Theoretically, any gene segment can be incorporated into the genome by providing a homologous template via the HDR pathway. Given that NHEJ is typically dominant in most mammalian cells, improving the efficiency of HDR is crucial to maximizing the power of gene‐editing platforms.^9^, ^10^ Therefore, knowing the mechanism and characteristics of diverse genome‐editing technologies, along with choosing suitable gene‐editing agents, contributes to reaching our aspiration of performing genetic change precisely (various gene‐editing technologies have been summarized in Table 1).

**TABLE 1:** Comparison between different gene‐editing agents

Gene‐editing technology	Clustered regularly interspaced short palindromic repeat (CRISPR) and CRISPR‐associated (Cas; CRISPR‐Cas) system	Base editors	Primer editors	Zinc finger nucleases (ZFNs)	Transcription activator‐like effector nucleases (TALENs)
Component for sequence recognition	Single guide (sgRNA) or CRISPR RNAs (crRNA)/ trans‐activating crRNA	sgRNA	sgRNA	Zinc finger proteins	Transcription activator‐like effectors (TALE)
Element for nucleic acid cleavage	Cas endonucleases (e.g.: SpCas9, Cas12a)	None	Cas9 nickase domain	FokI endonucleases	FokI endonucleases
Restrictions of target site	Adjacent to protospacer‐adjacent motifs (PAMs)	Adjacent to PAMs, ssDNA R‐loop	Adjacent to PAMs	Avoid non‐G rich sites	TALEN monomer located 5′‐end only recognizes T
length of target site	About 23 bp	> 17 bp	About 30 bp	(9–18 bp)*2	(12–20 bp)*2
Size	Cas nuclease > 2 kb,	∼5.3 kb	∼6.4 kb	∼1 kb*2	∼3 kb*2
Advantages	Simple design and preparation, low cytotoxicity, high specificity, multiple gene editing	Reduce off‐target effects, single base editing without double‐strand breaks and homology‐directed repair	Multiple edit capability: single base editing, insertion and deletion of multiple bases	Large recognition range, moderate editing efficiency	Easy to design, high specificity
Disadvantages	Potential off‐target toxicity and on target mutation, low editing efficiency	The restriction of PAM, less efficiency	Potential indels mutation, less efficiency, immunogenicity	Cytotoxicity, potential off‐target toxicity, inefficient cutting capability	Cytotoxicity, complex assembly, high production cost

*Note*: SpCas9, CRISPR‐Cas9 from *Streptococcus pyogenes*.

### 2.1 CRISPR‐Cas nucleases: A robust and versatile genome‐editing tool

In nature, the various CRISPR‐Cas systems discovered in bacteria and archaea are adaptive immune mechanisms that silence foreign nucleic acids to resist invasion by the pathogen.^11^, ^12^, ^13^ In brief, once Cas effectors assemble with guide RNA molecules containing spacers, the complex can bind and cleave specific sequences near the protospacer adjacent motif (PAM).^11^, ^14^ This mechanism inspired researchers to reprogram the DNA‐recognizing capability of the CRISPR‐Cas system by using different nucleases and guide RNA. Generally, CRISPR‐Cas systems are classified into two groups (six types and more than 20 subtypes) according to their components and mechanisms.^15^ For instance, class 1 CRISPR‐Cas systems, including Type I, Type III, and Type IV, can cleave nucleotide sequences depending on multiprotein complexes.^16^ In contrast, class 2 systems, which are subdivided into Type II, Type V, and Type VI, use single Cas effectors to achieve DNA cleavage.^17^, ^18^ Because of their unique programmability and structural advantage, class 2 systems have been widely used in multiple genome‐editing applications. Among the class 2 CRISPR‐Cas systems, Cas9, Cas12, and Cas13 are currently the research focus.

#### 2.1.1 CRISPR‐Cas9 system

**FIGURE 2:**
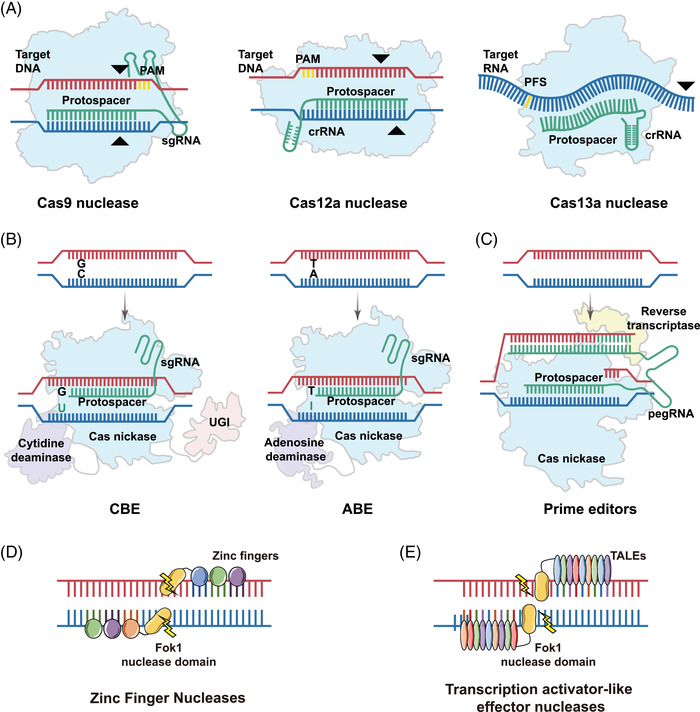
General overview of the primary gene‐editing tools. Schematic diagram of various gene‐editing tools. (A) Three frequently used clustered regularly interspaced short palindromic repeat (CRISPR) and CRISPR‐associated (Cas) nucleases: Cas9, Cas12a, and Cas13a. (B) Cytosine base editors (CBEs) are composed of the Cas9 nickase (Cas9n), a cytidine deaminase, and a uracil glycosylase inhibitor (UGI). Adenine base editors (ABEs) are composed of the Cas9n and engineered adenosine deaminase. (C) The prime editor consists of Cas nickase and reverse transcriptase. (D) and (E). Two protein‐based DNA targeting agents. Zinc finger nucleases (ZFNs) contain a zinc finger motif and the FokI restriction endonuclease. Transcription activator‐like effector nucleases (TALENs) have catalytic FokI endonucleases fused to a DNA‐binding domain TAENs

The natural CRISPR‐Cas9 systems are Type‐II CRISPR systems and are composed of DNA endonucleases and two RNA modules.^19^ A blunt DSB could be generated by this ribonucleoprotein (RNP) complex (Figure 2): CRISPR RNAs (crRNAs) paired with trans‐activating crRNAs (tracrRNAs) guide nucleases to target specific loci and facilitate complex formation; then, endonucleases are responsible for cutting nucleic acids.^13^ To optimize their operability, the crRNA and tracrRNA were integrated into a single guide RNA (sgRNA).

The characteristics of various Cas9 systems, including the PAM specificity,^20^ spacer length, endonuclease activity, and guide RNA architecture, directly determine the precise recognition and cleavage process.^21^ For example, CRISPR Cas9 from *Streptococcus pyogenes* (SpCas9) was the first reported Cas9 variant that was tested for manipulating genetic sequences in vitro and in mammalian cells.^22^, ^23^ For SpCas9 to perform normal functions, it is required for the user to choose an appropriate target location adjacent to the PAM 5′‐NGG (N represents any nucleotide) and design a 20 nt spacer within the sgRNA or crRNA/tracrRNA pair as well as an active nuclease.^22^, ^24^ In practice, scientists have discovered and developed many Cas9 variants to expand the scope of gene‐editing applications.^25^, ^26^, ^27^, ^28^, ^29^ These Cas9 orthologs have shown diverse characteristics from SpCas9, such as smaller protein size (*Staphylococcus aureus* Cas9 contains 1053 amino acids, *Campylobacter jejuni* Cas9 contains 984 amino acids)^30^, ^31^, ^32^ and abundant PAM sequences that broaden the target scope (*S. thermophilus* Cas9 recognizes the PAM 5ʹ‐NNAGAAW, and W represents A or T).^33^, ^34^

#### 2.1.2 CRISPR‐Cas12 nucleases

CRISPR‐Cas12 effectors from the Type‐V CRISPR system are also RNA‐guided endonucleases. Several innate features distinguish Cas12 from CRISPR‐Cas9 systems (Figure 2).^18^, ^35^ For instance, the recognition site of most Cas12 systems is located downstream of the PAM sequence, and the cleavage site within the protospacer is farther from the PAM. In addition, many Cas12 variants usually produce a staggered nucleic acid incision with a 4–5 nt overhang downstream of the PAM sequence by using a single crRNA.^36^ The first engineered Cas12 nuclease named Cas12a (formerly Cpf1) has been applied to modify the genome information in human cells.^36^, ^37^ The Cas12a effector inherently possesses the capacity to generate self‐required crRNA from crRNA arrays without needing tracrRNA.^38^ This unique advantage means that this system has the potential to simplify multiplexed gene editing since scientists simply need to design multiple crRNA arrays to achieve this goal.^38^, ^39^ Within Cas12a family proteins, two orthologs from *Acidaminococcus* spp. (AsCpf1) and *Lachnospiraceae* spp. (LbCpf1) have been shown to display gene‐editing activity in human cells by recognizing the T‐rich PAM (5′‐TTTV).^40^, ^41^ In addition to targeting and cutting DNA, some Cas12 variants (such as Cas12 g) can cleave the RNA when guided by a single crRNA.^42^, ^43^ The discovery of Cas12‐family endonucleases has propelled technical innovation in genome editing.

#### 2.1.3 CRISPR‐Cas13

After a series of bioinformatic analyses and computational efforts using microbial metagenomic database, some CRISPR‐Cas13 variants were identified by Feng Zhang and colleagues, including Cas13a (formerly named C2c2), Cas13b, Cas13c, and Cas13d.^44^, ^45^ Similar to Cas9 or Cas12 systems, Cas13 systems possess programmable nuclease activity and have the potential to be developed as RNA editing tools. The Cas13a effectors from the class 2 Type VI system are RNA‐guided RNA endonucleases that have been optimized and show activity in manipulating RNA (Figure 2).^46^ Recently, specific orthologs of Cas13, including Cas13b and Cas13d, were leveraged for RNA knockdown and manipulation and exhibited stability and high efficiency in RNA editing.^47^, ^48^ In addition to RNA knockdown and editing, Cas13 systems have been reformed for nucleic acid detection.^49^ The Cas13 protein will cleave any single‐stranded RNA in its vicinity after binding and cutting the targeted sequence.^50^ Based on the collateral cleavage activity of Cas13‐family endonucleases, Zhang et al. established a diagnostic platform termed specific high‐sensitivity enzymatic reporter unlocking (SHERLOCK).^51^, ^52^ Compared with the traditional nucleic acid detection method quantitative polymerase chain reaction (qPCR), this diagnostic technology can detect RNA or DNA within 10–15 min with great sensitivity.^53^ At present, more Cas13 orthologs have been discovered and tested for other applications, such as transcriptome regulation and RNA visualization in live cells.^54^

### 2.2 Base editors: The pearl in the crown of gene editing

Modest gene mutations, even a single nucleotide variation, may trigger the occurrence of genetic disease in humans.^55^ Currently, many genome‐editing tools have been applied to correct genetic mutations. Of these cases, most strategies must generate DSBs and depend on the HDR pathway. However, common single base modification, which depends on the DNA repair pathway induced by DSBs, is far from satisfactory.^56^, ^57^

To solve these issues, two different types of base editors based on CRISPR‐Cas have been developed for precise base manipulation. One is cytosine base editors (CBEs), and the other is adenine base editors (ABEs).^58^ These tools were able to install point mutations without DSB through the single‐strand repair pathway (Figure 1).^59^

#### 2.2.1 CBEs install C•G‐to‐T•A mutations

The basic components of CBEs include a personalized sgRNA and a multifunctional fusion protein that consists of catalytically deficient CRISPR‐Cas nuclease and single‐stranded DNA cytidine deaminase (Figure 2).^59^ According to guidance by sgRNA, Cas effectors can bind the target sequence and create an ssDNA R‐loop without generating DSBs. Generally, the editing window is a short fragment of exposed ssDNA (positions 4–8 are located in the protospacer if positions 21–23 are PAM). The cytidine deaminases are able to complete the transition point mutations (cytosines are converted to uracils) on the noncomplementary strand.^60^

To date, scientists have created multiple versions of CBEs to gain greater efficiency and safety. The first‐generation base editor, termed BE1, was formed using the cytidine deaminase enzyme APOBEC1 (apolipoprotein B mRNA editing enzyme, catalytic polypeptide‐1) from *Rattus norvegicus* and a dead Cas9 (dCas9) without endonuclease activity.^61^ However, the mismatched base pair (U•G) is rectified through the base excision repair pathway, which may result in poor BE1 editing effectiveness in mammalian cells.^62^, ^63^ To boost the base editing efficiency, the second‐generation base editor BE2 was developed by adding an extra uracil glycosylase inhibitor (UGI).^61^, ^64^ Because of the UGI, the U•G intermediate could remain in vivo longer to obtain stable base editing outcomes.^65^ To further increase the editing efficiency, the third version of the base editor (BE3, also described as CBE) replaced dCas9 with Cas9 nickase (Cas9n).^59^ Once the nondeaminated strand has been cleaved, the activated cellular repair mechanism will use the U‐containing DNA strand as a template for fixing the gap. Additionally, many variants of BE3 have been engineered to improve the editing precision by narrowing the editing window, namely, variants such as YE1‐BE3, YE2‐BE3, and YEE‐BE3.^66^, ^67^ Most recently, the fourth‐generation base editor BE4, which possesses two UGI proteins, was created for superior editing outcomes.^68^, ^69^

#### 2.2.2 ABEs install A•T‐to‐G•C mutations

The initial design of ABEs was a Cas9n tethered to an adenosine deaminase (ADA). As originally conceived, ABE can deaminate adenine within the R‐loop into inosine, which was eventually restored to guanine.^58^ However, the natural ADA cannot convert adenosine within ssDNA to inosine. Therefore, researchers have tried to reform the tRNA‐specific ADA (ecTadA) from *Escherichia coli* using directed evolution. After complex protein engineering, the desired mutation was discovered and fused to Cas9n. The final architecture of ABEs was composed of a heterodimeric ADA (ecTadA‐Tad*) and a Cas9n. This version was named ABE7.10, and it is the most commonly used base‐editing tool.^58^ The discovery of ABE7.10 offers the possibility of extending the range of base editing. Similar to CBEs, ABEs have been continuously upgraded to pursue superior editing effectiveness. For instance, ABEmax with several nuclear localization signal peptides has been developed based on ABE7.10.^68^

### 2.3 Prime editors: A potent new base modification technology

Four transition point mutations (A→G, T→C, C→T, and G→A) have been implemented by using CBEs and ABEs without reliance on the HDR pathway. However, the implementation of base transversions and the other eight types of point mutations (A→C, A→T, T→A, T→G, C→A, C→G, G→C, and G→T) require novel technology. In 2019, Liu's lab reported on a pioneering gene‐editing tool called prime editor, which could not only install all possible types of DNA point mutations but could also induce small deletions or insertions without donor DNA templates.^70^ The current prime editor consists of a prime editing guide RNA (pegRNA) and a Cas9n fused to engineered reverse transcriptase (Figure 2). The protein complex guided by pegRNA nicks the target DNA strand and mediates subsequent reverse transcriptional reactions. In fact, the pegRNA is a modified sgRNA that has an extra RNA sequence at the 3′ end.^70^ This guide RNA containing the desired gene fragment is responsible for recognizing the target sequence and serves as a template for subsequent reverse transcription. In brief, the DNA single‐strand nicked by Cas9n could use the pegRNA as the template to execute reverse transcription. After the reaction, the mutations are placed at the target site, generating a 5′ DNA flap and a 3′ DNA flap containing the edited sequence. Last, the 5′ DNA flaps were generally removed by the cellular DNA repair pathway.^71^ Compared with other genome‐editing tools, prime editors have advanced in some respects, including a more extensive application range, as well as better editing accuracy and safety with few byproducts.^60^ The superprecise CRISPR tool enhances our ability to manipulate genetic information and has the potential to promote the development of gene therapy.^72^

### 2.4 ZFNs: The first generation of mature gene‐editing technology

ZFNs, which consist of zinc finger motifs and the FokI restriction endonuclease, are considered one of the earliest gene‐editing tools (Figure 2). Zinc finger proteins are responsible for recognizing and binding nucleotide sequences and are versatile transcription factors in eukaryotic organisms.^73^ Each zinc finger protein consists of approximately 30 amino acids and forms a ββα structure, with a Cys2‐His2 domain interacting with the zinc atom.^74^ According to the three‐dimensional cocrystal structure, the NDA recognition capacity of zinc finger proteins (ZFs) relies on the α‐helical reading head bound with 3 bp within the major groove of the double helix.^75^ The Type II restriction enzyme FokI derived from *Flavobacterium okeanokoites* requires dimerization to complete DNA cleavage, and the cutting ability is independent without side effects.^76^, ^77^ Generally, dimerized ZFNs can recognize 18–36 bps of the target sequence and generate a staggered DSB with a 5′ overhang. In principle, ZFNs can target any desired gene sequence by using various zinc finger proteins. In addition, the DNA binding specificity also depends on the number of fingers and the target location. However, the production of highly specific ZFNs is far from satisfactory since the assembly and optimization procedure required a great deal of time and effort. To extend the range of DNA recognition, many strategies, such as “two‐finger modules^78^” and the “open system,”^79^ have been established to reform and optimize the structure and function of ZFNs. For example, scientists obtained new ZFN architectures with a larger recognition range by using new linkers derived from a cleavage‐based bacterial selection system.^80^

Many advantages, including low immunogenicity and appropriate gene size, have prompted the widespread use of ZFNs as gene‐editing tools for multiple applications. Unfortunately, the further large‐scale application and popularization of ZFNs were hindered by some technical barriers. Admittedly, the synthesis and assembly of zinc finger proteins are time‐consuming and require technical expertise. In addition, the related technology patents belong to several agencies, which hinders the widespread use of ZFNs.

### 2.5 TALENs: A flexible protein‐based editing system

Because of the shortage of publicly available resources, it is difficult to construct ZFNs. To overcome these technical limitations, a protein‐based DNA editing platform known as TALENs was established. TALENs contain a catalytic FokI endonuclease fused to DNA‐binding domain TALEs (Figure 2). Natural TAL effectors have been discovered in plant pathogenic *Xanthomonas* bacteria, and they change the transcription in the host cells.^81^ The DNA targeting capacity of TALEs depends on highly conserved tandem arrays, which consist of 10–30 repeats. Individual TALE repeats that contain approximately 33–35 amino acids could target a single nucleotide accurately. The architecture of a single TALE includes two helix bundles and a loop. Notably, two hypervariable residues (residues 12 and 13) within the loop structure, which is currently named the repeat variable di‐residue (RVD), determine the specificity and affinity of each effector.^82^ The RVD can not only ensure the stability of the loop structure but can also target specific nucleotides.^83^ To date, four identified RVDs, HD (HD represents the residules of histidine and aspartic acid, specifies cytosine), NG (NG represents the asparagine and glycine residues, specifies thymine), NI (NG represents the asparagine and isoleucine residues, specifies adenine), and NN (NN means two asparagine residues, specifies guanine or adenine), have been widely employed to generate the desired TALENs for multiple gene‐editing applications.

Similar to ZFNs, the platform based on TALENs can be reprogrammed to bind arbitrary sequences by rearranging TALE repeats, but the complicated assembly procedure limits its promotion. To accelerate the assembly process, several strategies have been implemented, including the Golden Gate cloning system, restriction enzyme and ligation technology, fast ligation‐based automatable solid‐phase high‐throughput system, and ligation‐independent cloning technology.^84^ In addition to their programmability, TALENs have several obvious advantages: simplified design and construction and a flexible range of recognition. For instance, dimerized TALENs are usually engineered to bind 36‐bp sequences or even longer. Of course, there are some disadvantages that must be solved. The large size of TALENs (approximately 3 kilobases [kb]) may cause delivery difficulties. During the delivery of TALENs, the repeats are susceptible to rearrangements,^85^ which carries a risk of genetic manipulation.

## 3 GENOME EDITING FOR FUNDAMENTAL RESEARCH: DISEASE MODELLING AND DISEASE DIAGNOSIS

### 3.1 Targeted genome editing tools for disease modeling

The unprecedented ability to create a gene modification at a specific site has driven the development and upgradation of medical disease models. Numerous successful disease models have been established for further detailed pathologic studies.

#### 3.1.1 Cardiovascular disease (CVD) models

CVD is considered a critical public health issue because of its high mortality and morbidity.^86^ Most CVDs are inherited diseases characterized by monogenic mutations or a complex of heterozygous mutations.^87^ Carroll et al. created a Cas9 transgenic mouse model that only expresses a high level of Cas9 in the heart, but no evident burden was observed in vivo.^88^ Driven by adeno‐associated virus (AAV), sgRNA was delivered to the *Myh4* locus to induce an accurate and rapid depletion of cardio‐relevant genes and subsequently show severe cardiomyopathy. The fibrillin‐1 encoded by the*FBN1* gene is the fundamental component of the connective tissue matrix. Heterozygous mutations in *FBN1* often correlate with the phenotype of Marfan syndrome (MFS).^89^, ^90^ Using pigs as a human biomedicine model could promote the study of human diseases. Many medical studies require a suitable animal model that resembles humans to repeat experiments. For example, the porcine model is considered a more suitable candidate for investigating CVD because of its unique biological characteristics. Specifically, the anatomical, physiological, and genetic characteristics of pigs are similar to those of humans.^91^ Some porcine somatic cells have been modified by ZFNs to generate a cloned pig with a heterozygous *FBN1* mutant (+/Glu433AsnfsX98), which exhibited phenotypes (scoliosis, pectus excavatum, and structural damage in the aortic medial tissue) similar to those of patients with MFS.^92^ Based on a similar strategy, Yang et al. took advantage of ZFNs and electroporation to knock out the gene encoding peroxisome proliferator‐activated receptor‐gamma (*Ppar‐γ*) in primary porcine cells and then generated an invaluable Ppar‐γ knockout (KO) porcine model for studying CVD.^93^

#### 3.1.2 Metabolic disease models

Metabolic disorders represent a dysfunctional state in the body, hampering the shifting of food energy into cell bioreactions.^94^ Leptin (*Lep)* and its corresponding receptor (*LepR*) are responsible for inhibiting fat synthesis and balancing energy metabolism.^95^ To elucidate their pivotal function in glycolipid metabolism, scientists have created multiple animal models, which are typically represented by obesity mutant mouse models and diabetes mutant mouse models.^96^ Using CRISPR‐Cas9 technology, Bao and colleagues established *LepR* knockout rats, which ultimately exhibited complications of obesity and diabetes.^97^ Furthermore, Chen et al. successfully installed diverse LepR mutations into mouse embryos by TALENs, generating several transgenic rats. Obesity, insulin resistance, and metabolic disorders were observed in some rats with a frame‐shifted or premature stop codon mutation. The study reported the first rat models for obesity research based on the Sprague Dawley strain.^98^ As the key regulator in the metabolism of glucose and lipids, cAMP (cyclic adenosine monophosphate)‐responsive element‐binding protein 3‐like 3 (CREB3L3) is predominantly expressed in the liver and intestine.^99^ To investigate the different functions of CREB3L3 within various tissues, some Japanese scholars invented liver‐ and small intestine‐specific CREB3L3 knockout mice through the CRISPR‐Cas9 system. With the help of these mouse models, these investigators provided a new understanding regarding the role of CREB3L3 in different metabolic pathways.^100^

#### 3.1.3 Models for neurodegenerative diseases (NDs)

NDs (such as Huntington's disease [HD], Alzheimer's disease [AD] and Parkinson's disease [PD]) have always been a threat to human health. Due to the deficiency in effective diagnostic and therapeutic schemes, the public health care system suffers from stress.^101^ In particular, the dearth of suitable disease models has hampered progress in the field. Currently, many in vitro and in vivo studies have demonstrated that the huntingtin (*HTT*) gene is closely related to the occurrence of HD.^102^, ^103^, ^104^ To elucidate the pathogenesis of HD, diverse animal models have been investigated through gene editing. After introducing truncated mutant *HTT* into HD140Q knock‐in mice through the CRISPR‐Cas system, investigators found that exon 1 *HTT* is a key pathogenic factor of HD.^105^ Furthermore, Yan et al. generated a gene‐engineered porcine model with full‐length mutant *HTT* using CRISPR‐Cas9 and somatic nuclear transfer technology. These large animal models recapitulated overt and selective neurodegeneration exhibited in HD patients, while pathological features can be germline‐transmitted to their progeny.^106^

The amyloid precursor protein (*APP*) gene has also been extensively studied in NDs, specifically AD.^107^ To obtain APP‐overexpressing cell lines, the partial coding regions of *APP* were incorporated into the genome of mouse fibroblast cells by ZFNs. These modified cells illustrate how APP mutants influence relevant signaling pathways to induce the onset of AD.^108^ TALENs were also used to create A673 V and A673T variants based on human‐induced pluripotent stem cells (iPSCs), which ultimately developed various levels of AD‐featured biomarkers.^109^ In addition, Paquet et al. installed both heterozygous and homozygous mutations of *A* *PP* or presenilin (PSEN) 1 in human iPSCs enabling the appearance of early AD symptoms.^110^

#### 3.1.4 Hereditary ocular diseases

In recent years, emerging gene‐editing technology has made it possible to establish hereditary eye disease models, which help to explain the pathological mechanisms and to identify pathogenic genes. The existing eye disease models are primarily concentrated on retinitis pigmentosa (RP), Leber congenital amaurosis (LCA), and retinoblastoma, among others.

Receptor expression enhancer protein 6 (*REEP6*) is highly expressed in rod photoreceptor cells, contributing to the formation of the endoplasmic reticulum.^111^ Several biallelic variants of *REEP6* have been identified in seven unrelated individuals affected with RP. Therefore, one of the verified missense mutants, which is known as *REEP6.1*, was integrated into the murine genome through CRISPR‐Cas9, inducing clinical manifestations of RP, such as retinal degeneration and the malfunction of rod photoreceptors.^112^ LCA, a congenital eye disease, can lead to retinopathy and early blindness.^113^ At present, numerous mouse models have been generated to support the functional exploration of all other LCA genes. For instance, some evidence has shown that *KCNJ13* mutants are closely linked to the early onset of LCA. Subsequently, different concentrations of spCas9RNA and sgRNA were delivered into zygotes using microinjection to generate a *KCNJ13* gene‐null mouse model. The results show that high concentration‐derived mice are more efficient and compatible with mimicking the pathogenesis of *KCNJ13*‐induced LCA and that the deficiency of *KCNJ13* causes photoreceptor degeneration.^114^

#### 3.1.5 Other diseases

Von Willebrand disease (VWD) is the most common bleeding disorder and is characterized by coagulation disorders.^115^ Hai et al. reported a convenient and efficient method for preparing VWD models. They took advantage of the CRISPR‐Cas9 platform to knock out the double allele of the *VWF* gene in pigs. This miniature pig model perfectly circumvents the current obstacle in which small rodents cannot highly recapitulate the disease hallmarks of human patients.^116^

Rett syndrome (RTT) is a serious disorder affecting children's neurodevelopment. Arrested growth predominantly occurs in females aged 6–18 months old. However, for males, RTT is fatal during the fetal period.^117^ Methyl‐CpG binding protein 2 (*MECP2*) is an X‐linked gene, the mutations of which correlate with the rise of the autism spectrum.^117^, ^118^ A research team from China employed TALENs to create the first *MECP2* mutation model based on rhesus and cynomolgus macaques. These models have shown almost identical embryonic lethality with human boys suffering from RTT. Additionally, when using the same gene‐editing system, this team generated another cynomolgus monkey model with mutagenesis of the RTT, which displayed stark abnormalities in physiology, behavior, and body structure.^119^

Thanks to the rapid advancement of genome‐editing technologies, numerous severe and refractory diseases also have available disease models for study, such as sickle cell disease (SCD),^120^ Niemann–Pick disease,^121^ and Duchenne muscular dystrophy.^122^ Progress in the genetic engineering area will further extend the application of animal models to elucidate the more sophisticated molecular mechanisms and raise entirely new prospects for therapeutic modalities.

### 3.2 New disease diagnostic tools based on the CRISPR‐Cas system

In many cases, efficient therapeutic intervention relies on the timely and accurate diagnosis of disease. The currently relevant application of gene editing has shown unique advantages in the diagnosis of diseases, especially cancer and viral infections.^123^, ^124^ The currently reported CRISPR‐Cas systems can be broadly divided into two classes and some subtypes, according to the nature of the effector protein complex.^15^, ^18^ Of these systems, the Class 2 CRISPR system, including Types II, V, and VI, is principally applied to develop novel diagnostic tools. For instance, one group made an attempt to create a CRISPR‐Cas9n‐mediated strand displacement amplification method (abbreviated as CRISDA). Multiple experimental results indicate that CRISDA has the potential to be a powerful diagnostic tool because of its single‐nucleotide specificity and versatility.^125^

One interesting application of the CRISPR‐Cas system is in the diagnosis of cancer. Cancer, as an important disease threatening human health, has always had a very high mortality rate. The important reason for mortality is that patients often miss the best time for treatment because of the shortage of early diagnostic approaches. The efficient early diagnosis of cancer is critical to making an effective intervention for disease progression and providing a better quality of life. In 2017, Gootenberg et al. proposed a brand‐new concept using CRISPR‐Cas13n‐mediated diagnostic techniques, which offers another ultrasensitivity and specificity option for testing RNA and DNA sequences.^49^, ^51^ The platform, SHERLOCK, offers a chance to screen disease‐causing mutations. Using this method, researchers found that cancer‐related *EGFR‐L858R* (EGFR, epidermal growth factor receptor) and BRAF‐V600E mutations with allelic fractions as low as 0.1% can be detected in cell‐free DNA fragments. In subsequent studies, scientists successfully detected *EGFR‐L858R* and *EGFR‐T790* M mutations in DNA fragments derived from non‐small cell lung cancer (NSCLC) patients.^126^ Inspired by SHERLOCK, investigators used Cas12a and recombinase polymerase amplification to establish another diagnostic tool named DNA endonuclease‐targeted CRISPR trans reporter (DETECTR), which has attomolar sensitivity and detects infectious viruses associated with cancer. Typically, human papillomavirus (HPV) 16 and HPV18, the two most pro‐oncogenic HPV species, can be identified and distinguished by DETECTR.^127^ In practical applications, the detection efficiency is satisfactory, and the detection rate fluctuates between 90% and 100%.^128^ Furthermore, a reusable electrochemical biosensor consisting of the CRISPR‐Cas13a system and a catalytic hairpin DNA circuit has been leveraged to screen tumor‐related RNA for the early diagnosis of NSCLC.^129^

Another interesting application of the CRISPR‐Cas system is in the diagnosis of infectious diseases. Most currently, global health care systems have been undermined to an unprecedented extent by the novel coronavirus (COVID‐19) pandemic, which is caused by severe acute respiratory syndrome coronavirus 2 (SARS‐CoV‐2).^130^, ^131^, ^132^ Accurate and rapid diagnosis will benefit patients to receive timely treatment. However, enzyme‐linked immunosorbent assat (ELISA) and real‐time or reverse transcription‐polymerase chain reaction (RT‐PCR) are common and classical molecular detection tools that require intricate equipment and fastidious work processes.^133^ It is imperative to establish a portable, accurate, and convenient diagnostic system for detecting infectious disease pathogens. Diagnostic CRISPR systems have outstanding superiority in terms of their ultrahigh sensitivity, portability, and specificity. For instance, the Cas12 and Cas13 nucleases have been reprogrammed to detect the nucleic acids of SARS‐CoV‐2.^134^, ^135^, ^136^

Viruses, whether DNA viruses or RNA viruses, are important pathogens of infectious diseases. RNA viruses that have been studied abundantly in recent years include dengue,^137^ Zika,^49^ and a recent spotlight of interest, SARS‐CoV‐2.^138^ For SARS‐CoV‐2 detection, the SHERLOCK and DETECTR detection systems mentioned above have been modified and upgraded to achieve higher efficiency in virus extraction,^139^ more flexibility in sensing capability, and a more streamlined mechanical process.^140^, ^141^ In 2022, Lu et al. reduced the detection time of CRISPR‐based assays while ensuring accuracy through a hybrid strategy, which includes adjusting the kinetics of Cas12a and using more flexible crRNA designs.^134^ In addition to RNA viruses, CRISPR/Cas‐based diagnostic methods can identify DNA viruses, such as BK virus (BKV), cytomegalovirus (CMV), and Epstein–Barr virus (EBV).^142^, ^143^ The researchers used the SHERLOCK system to detect BKV or CMV in serum samples and then verified the results by qPCR. The comparison results demonstrated that the two approaches have almost the same specificity. To date, the Food and Drug Administration (FDA) has authorized a CRISPR‐based COVID‐19 diagnostic tool for emergency use. The successful translational application of the CRISPR‐Cas system in COVID‐19 diagnosis indicated that this versatile technology has the potential to be applied to other severe epidemic disease diagnoses in the future.

## 4 CURRENT APPLICATION OF GENE EDITING TO THE TREATMENT OF HUMAN DISEASES

The ultimate aspiration pursued by researchers is to use gene‐editing technologies to treat human diseases without undesired side effects. The current gene‐editing techniques have provided a brilliant opportunity for precise intracellular gene manipulation, which can not only be used to induce mutations, corrections, or deletions but can also introduce foreign genes at specific sites.^144^, ^145^, ^146^ Additionally, precise genetic manipulation can efficaciously reduce the risk of insertion mutations in some cell therapies.^147^ Admittedly, novel therapies offer an attractive opportunity to save patients’ lives. In contrast, the consequence of gene editing is unknown and fraught with potential risks. Therefore, performing sufficient preclinical research is an important prerequisite for promoting gene editing for clinical application. As described in the previous section, advances in disease modeling and diagnostic techniques have driven the development of preclinical research on gene‐editing therapeutics.^148^, ^149^ According to these unique advantages and the promising outcomes of preclinical studies, various gene‐editing agents have been leveraged to treat human diseases in clinical trials.

**FIGURE 3:**
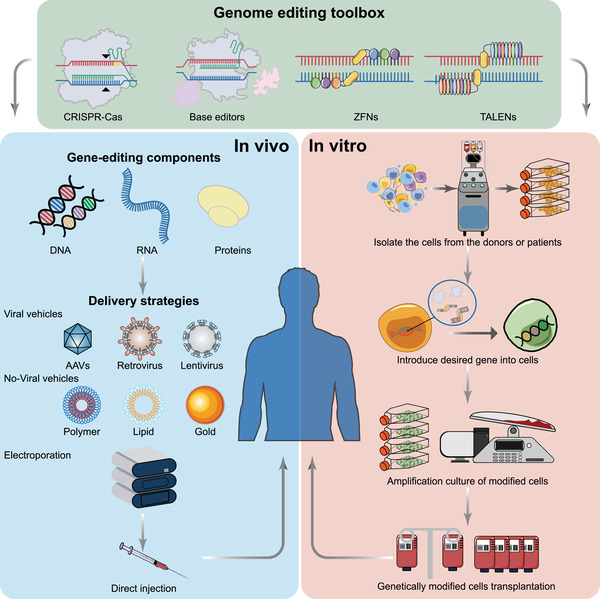
Ex vivo and in vivo therapeutic gene‐editing strategies. Gene‐editing therapeutics consist of two modes. The in vivo gene‐editing strategy (left) is straightforward. The vectors containing desired gene cargoes and editing machinery are injected into the targeted tissues or organs to perform gene editing. The treatment process of ex vivo gene‐editing therapy (right) can be roughly divided into the following four steps: (1) separate the required cells from the donor and culture them in vitro; (2) use an appropriate gene‐editing platform to modify the cell genome; (3) expand and cultivate the edited cells in vitro; and (4) inject the edited cells back into the patient for treatment. AAV, adeno‐associated virus; TALENs, transcription activator‐like effector nucleases; ZFNs, zinc finger nucleases

Generally, the strategies of gene‐editing therapeutics contain two modes, including in vivo and ex vivo strategies (Figure 3). To date, numerous pre‐ and clinical studies of gene editing have been scattered across various countries. In this section, we will provide a comprehensive overview of this clinical information and primarily discuss the progress in therapeutic gene editing in clinical trials (relevant clinical trial information is summarized in Table 2). Additionally, we also introduce some interesting preclinical studies and highlight several current novel therapeutic strategies and concepts.

**TABLE 2:** Current clinical trials applying genome editing for treating human diseases

Therapeutic area	Disease application	Edited cells	Target	Number of included patients	Platform	Delivery	Clinical phase	Identifier	Status
Cancer	B‐cell acute lymphoblastic leukemia (B‐ALL)	T cells	TRAC and CD52	25	TALEN	mRNA electroporation	I	NCT02746952	Completed
	B‐ALL	T cells	TRAC and CD52	60	TALEN	mRNA electroporation	I	NCT04150497	Recruiting
	Relapsed/refractory large B‐cell lymphoma	T cells	TRAC and CD52	74	TALEN	mRNA electroporation	I	NCT03939026	Active, not recruiting
	Relapsed/refractory follicular lymphoma								
	ALL in relapse	T cells	Endogenous inactivate hematopoietic progenitor kinase 1	40	CRISPR	mRNA electroporation	I	NCT04037566	Recruiting
	ALL in refractory								
	B‐cell lymphoma								
	B‐cell malignancy	T cells	β2M and T‐cell receptor (TCR)	143	CRISPR‐Cas9	Electroporation	I/II	NCT04035434	Recruiting
	Non‐Hodgkin lymphoma								
	B‐cell lymphoma								
	B‐cell leukemia	T cells	β2M and TCR	80	CRISPR‐Cas9	Electroporation	I/II	NCT03166878	Recruiting
	B‐cell lymphoma								
	B‐cell leukemia	T cells	TRAC	80	CRISPR‐Cas9	Electroporation	I/II	NCT03398967	Recruiting
	B‐cell lymphoma								
	B‐cell non‐Hodgkin lymphoma	T‐cell	TRAC and PDCD1	50	chRDNA CRISPR	Plasmid transfection	I	NCT04637763	Recruiting
	B‐ALL	T‐cell	TRAC and CD52	10	CRISPR‐Cas9	Lentiviral vector	I	NCT04557436	Recruiting
	B‐cell non‐Hodgkin lymphoma	T cells	PD‐1	50	CRISPR	/	I	NCT04637763	Recruiting
	Relapsed/refractory B‐ALL	T cells	CD52 and TRAC	10	CRISPR	/	I	NCT04925206	Recruiting
	T‐cell lymphoma	T cells	β2M and TCR	45	CRISPR‐Cas9	Electroporation	I	NCT04502446	Recruiting
	T‐cell acute lymphoblastic leukemia	T cells	CD7	21	CRISPR‐Cas9	/	I	NCT03690011	Recruiting
	T‐cell acute lymphoblastic lymphoma								
	T‐non‐Hodgkin Lymphoma								
	Relapsed/refractory CD5^+^ hematopoietic malignancies	T cells	CD5	18	CRISPR‐Cas9	Electroporation	I	NCT04767308	Not yet recruiting
	Acute myeloid leukemia	T cells	TRAC and CD52	/	TALEN	mRNA electroporation	I	NCT04106076	Withdrawn
	Relapsed/refractory acute myeloid leukemia	T cells	TRAC and CD52	65	TALEN	mRNA electroporation	I	NCT03190278	Recruiting
	Acute myeloid leukemia	T cells		54	CRISPR‐Cas9	/	I/II	NCT05066165	Recruiting
	Relapsed/refractory multiple myeloma (MM)	T cells	TRAC and CD52	18	TALEN	mRNA electroporation	I	NCT04142619	Recruiting
	Acute myeloid leukemia	Hematopoietic stem and progenitor cell	CD33	33	CRISPR‐Cas9	/	long‐term follow‐up (LTFU) study	NCT05309733	Recruiting
	MM	T cells	β2M and TCR	80	CRISPR‐Cas9	Electroporation	I	NCT04244656	Recruiting
	MM	T cells	TCR PDCD1	3	CRISPR	mRNA electroporation	I	NCT03399448	Terminated (Sponsor has terminated trial to pursue other targets.)
	Melanoma synovial sarcoma								
	Myxoid/round cell liposarcoma								
	Metastatic non‐small cell lung cancer	T‐cell	PDCD1	12	CRISPR‐Cas9	Plasmid Electroporation	I	NCT02793856	Completed
	Gastrointestinal epithelial cancer	TIL	Immune checkpoint CISH	20	CRISPR‐Cas9	Plasmid	I/II	NCT04426669	Recruiting
	Gastrointestinal neoplasms								
	Gastrointestinal cancer								
	Colorectal cancer								
	Pancreatic cancer								
	Gallbladder cancer								
	Colon cancer								
	Esophageal cancer								
	Stomach cancer								
	Solid tumor	T cells	PDCD1	10	CRISPR‐Cas9	Protein and mRNA electroporation	I	NCT03747965	Unknown
	Mesothelin‐positive multiple solid tumors	T cells	TCR PDCD1	10	CRISPR‐Cas9	Protein and mRNA electroporation	I	NCT03545815	Recruiting
	Renal cell carcinoma	T cells	β2M and TCR	107	CRISPR‐Cas9	Electroporation	I	NCT04438083	Recruiting
	Esophageal cancer	T cells	PDCD1	16	CRISPR‐Cas9	Electroporation	NA	NCT03081715	Completed
	Neurofibromatosis type 1 tumors of the central nervous system	Induced pluripotent stem cells (iPSCs)	NF1	20	CRISPR‐Cas9	DNA	NA	NCT03332030	Suspended (Suspended due to cessation of funding)
	Advanced hepatocellular carcinoma	T cells	PDCD1	10	CRISPR‐Cas9	DNA	I	NCT04417764	Recruiting
	Invasive bladder cancer stage IV	T cells	PDCD1	Withdrawn	CRISPR‐Cas9	DNA	I	NCT02863913	Withdrawn
	Metastatic renal cell carcinoma	T cells	PDCD1	Withdrawn	CRISPR‐Cas9	DNA	I	NCT02867332	Withdrawn
	Metastatic renal cell carcinoma	T cells	PDCD1	Withdrawn	CRISPR‐Cas9	DNA	I	NCT02867332	Withdrawn
	Stage IV gastric carcinoma	Cytotoxic T lymphocyte cells	PDCD1	20	CRISPR‐Cas9	Electroporation	I/II	NCT03044743	Unknown
	Stage IV nasopharyngeal carcinoma								
	T‐cell lymphoma								
	Stage IV adult Hodgkin lymphoma								
	Stage IV diffuse large B‐cell lymphoma								
	Solid Tumor, EGFR overexpression	T cells	TGF‐β receptor Ⅱ	30	CRISPR‐Cas9	/	I	NCT04976218	Recruiting
Hematological diseases	Hemophilia B	Hepatocytes	Clotting factor XI gene	1	ZFN	Adeno‐associated virus (AAV)	I	NCT02695160	Terminated
	Sickle cell disease (SCD)	Autologous hematopoietic stem cells (HSCs)	BCL11A	8	ZFN	mRNA	I/II	NCT03653247	Recruiting
	Transfusion‐dependent beta‐thalassemia	Autologous CD34^+^ hematopoietic stem/progenitor cells	BCL11A	6	ZFN	mRNA	I/II	NCT03432364	Active, not recruiting
	Thalassemia	iHSCs	HBB gene	12	CRISPR‐Cas9	/	Early I	NCT03728322	Unknown
	Beta‐thalassemia Thalassemia genetic	CD34^+^ human hematopoietic stem and progenitor cells	BCL11A gene	45	CRISPR‐Cas9	Electroporation	I/II	NCT03655678	Active, not recruiting
	Inborn hematologic diseases								
	Hemoglobinopathies								
	Transfusion dependent beta‐thalassemia	CD34^+^ human hematopoietic stem	BCL11A gene	8	CRISPR‐Cas9	Electroporation	I	NCT04925206	Recruiting
	SCD	CD34^+^ human hematopoietic stem and progenitor cells	BCL11A gene	45	CRISPR‐Cas9	Electroporation	I/II	NCT03745287	Active, not recruiting
	SCD	CD34^+^ human hematopoietic stem	HBB gene	15	CRISPR‐Cas9	Adenoviral vector	I/II	NCT04819841	Recruiting
	SCD	CD34^+^ human hematopoietic stem	β‐globin gene	9	CRISPR‐Cas9	Electroporation	I/II	NCT04774536	Not yet recruiting
	Transfusion dependent β‐thalassemia	CD34^+^ Human Hematopoietic Stem and Progenitor Cells (hHSPCs)	BCL11A gene	8	CRISPR‐Cas9	electroporation	I	NCT04925206	Recruiting
	Severe SCD	CD34^+^ hHSPCs	BCL11A gene	12	CRISPR‐Cas9	/	III	NCT05329649	Not yet recruiting
	Severe SCD	CD34^+^ HSCs		15	CRISPR‐Cas9	/	I/II	NCT04819841	Recruiting
Viral infections	Human immunodeficiency virus (HIV) infection	Autologous CD4^+^ T cells	C‐C chemokine receptor type 5 (CCR5)	12	ZFN	Adenoviral vector	I	NCT00842634	Completed
	HIV infection	Autologous CD4+T cells	CCR5	21	ZFN	Adenoviral vector	I/II	NCT01252641	Completed
	HIV infection	Autologous CD4+T cells	CCR5	19	ZFN	Adenoviral vector	I	NCT01044654	Completed
	HIV infection	Autologous CD4+T cells	CCR5	8	ZFN (SB‐728mR)	Electroporation	I/II	NCT02225665	Completed
	HIV infection	Autologous CD4+T cells	CCR5	14	ZFN (SB‐728mR)	Electroporation	I	NCT02388594	Completed
	HIV infection	HSPCs	CCR5	12	ZFN	Electroporation	I	NCT02500849	Active, not recruiting
	HIV infection	T cells	CCR5	12	ZFN	mRNA	I	NCT03617198	Active, not recruiting
	HIV infection	T cells	CCR5	30	ZFN	Adenoviral vector	I/II	NCT03666871	Active, not recruiting
	HIV infection	HIV infected cells and tissues	Remove viral DNA from the genomes of cells and tissues	9	CRISPR‐Cas9	Adenovirus‐associated virus vector serotype 9 (AAV9)	I	NCT05144386	Recruiting
	HIV infection	HIV infected cells and tissues	Remove viral DNA from the genomes of cells and tissues	9	CRISPR‐Cas9	AAV9	LTFU study	NCT05143307	Enrolling by invitation
	Human papillomavirus (HPV)‐related malignant neoplasm	Tumor cells	HPV16 E6/E7	40	TALEN	Roche plasmid transfection	I	NCT03226470	Recruiting
	HPV‐related malignant neoplasm	Tumor cells	HPV16 E6/E7	60	TALEN and CRISPR‐Cas9	Plasmid transfection	I	NCT03057912	Unknown
	HPV‐related malignant neoplasm	Tumor cells	HPV16 E6/E7	20	ZFN	Plasmid transfection	I	NCT02800369	Unknown
	Coroavirus disease (COVID‐19)	T cells	PD‐1and ACE2	16	CRISPR‐Cas9	/	I/II	NCT04990557	Not yet recruiting
	Refractory herpetic viral keratitis	Herpes simplex virus type I	Herpes simplex virus type I	6	CRISPR‐Cas9	/	I/II	NCT04560790	Active, not recruiting
Metabolic disorders	Mucopolysaccharidosis II	Hepatocytes	IDS gene	9	ZFN	AAV	I/II	NCT03041324	Terminated
	Adenosine deaminase (ADA) deficiency severe combined immunodeficiencies	Autologous HSPCs	ADA gene	36	/	Lentivirus	I/II	NCT01380990	Completed
	Type 1 diabetes mellitus	Pancreatic endoderm cells		10	CRISPR‐Cas9	/	I	NCT05210530	Recruiting
	Transthyretin (TTR)‐related (ATTR) familial amyloid polyneuropathy	Hepatocytes	TTR protein gene	74	CRISPR‐Cas9	Lipid nanoparticles (LNPs)	I	NCT04601051	Recruiting
	Transthyretin‐related (ATTR) familial amyloid cardiomyopathy								
	Wild‐type transthyretin cardiac amyloidosis								
Ocular disorder	Blindness Leber congenital amaurosis 10 vision disorders	Photoreceptor cells	CEP290	18	CRISPR‐Cas9	AAV	I/II	NCT03872479	Recruiting
Others	Kabuki syndrome 1	Mesenchymal stem cells	KMT2D	8	CRISPR‐Cas9	/	NA	NCT03855631	Completed
	Rubinstein–Taybi syndrome	iPSCs	CREBBP	154	CRISPR‐Cas9	/	NA	NCT04122742	Recruiting
	Hereditary angioedema	Hepatocytes	KLKB1	55	CRISPR‐Cas9	LNPs	I/II	NCT05120830	Recruiting

*Note*: All data in Table 2 were collected from ClinicalTrials.gov, which is a resource provided by the US National Library of Medicine (https://clinicaltrials.gov).

### 4.1 Cancer immunotherapy

There are currently more than 70 clinical trials in the NIH clinical trial database that involve gene editing‐mediated therapy, including the use of ZFNs, TALENs, or CRISPR/Cas, and nearly 50% of these trials are related to neoplasms. Cancer immunotherapy is widely considered one of the most significant advances in biological research in recent years. The development of adoptive T‐cell therapy is the most prominent. The advent of cytotoxic T lymphocyte, T‐cell receptor transgenic (TCR) T cells, chimeric antigen receptor T cells (CAR‐T) and other therapeutic products has alleviated the symptoms and prolonged the lifetimes of patients with cancer.

In the field of gene‐editing‐mediated cancer immunotherapy, as many as 19 clinical trials have focused on CAR‐T therapy, including hematological and solid tumors. It is well known that CAR‐T‐cell therapy has shown convincing evidence during clinical trials for the treatment of hematological malignancies.^150^ However, as a customized therapeutic product, the widespread use of CAR‐T cells is limited. Currently, the development of universal CAR‐T (UCAR‐T) products is a major trend in the area of T‐cell therapy.^151^ The emergence of UCAR‐T cells expands the range of application, improves the feasibility of application, and may also reduce the cost of treatment. Even if the program is still facing technical barriers, there is no doubt that UCAR‐T therapy will be a key development direction in the future. As a leader in UCAR‐T, the French company Cellectis has developed an allogeneic CAR‐T technology platform based on TALEN gene‐editing technology. Cellectis Inc. has invented four “off the shelf” CAR‐T products by inactivating the TCR and CD52 genes: UCART22 targets CD22 to treat B‐cell acute lymphoblastic leukemia (B‐ALL; NCT04150497); UCART123 targets CD123 to treat acute myeloid leukemia (NCT03190278); UCARTTCS targets the CS1 antigen to treat relapsed/refractory multiple myeloma (MM; NCT04142619); and UCART19 is the first UCAR‐T product for treating B‐ALL (NCT02746952; NCT02808442). ALLO‐501A, a UCAR‐T product developed by Allogene Therapeutics using a similar concept, has already initiated clinical trials for treating B‐cell lymphoma (NCT04416984). Another company, CRISPR Therapeutics, has invented three UCAR‐T therapies based on the CRISPR gene‐editing system, namely, CTX110, CTX120, and CTX130. Unlike Cellectis Inc., CRISPR Therapeutics chooses to destroy β2M and TCR loci to reduce the risk of rejection. CTX110 is a CD19‐specific CAR‐T that is primarily used to treat B‐cell malignancy (NCT04035434). CTX120 kills MM cells by recognizing BCMA (NCT04244656), while CTX130 is used to treat CD70‐expressing T‐cell lymphoma and renal cell carcinoma (NCT04502446; NCT04438083). In addition, some researchers designed “off the shelf” CAR‐T cells against CD19 or mesothelin to treat B‐cell hematological or solid tumors (NCT03166878; NCT03545815).

Apart from UCAR‐T therapy, gene modification technology can be used to disrupt endogenous genes to improve the effectiveness of CAR‐T cells. For example, in a Phase I clinical trial, researchers used the CRISPR system to inactivate hematopoietic progenitor kinase 1, an intracellular negative regulator of T‐cell proliferation and signal transduction, to enhance the effect of CD19 CAR‐T cells (NCT04037566). In another Phase I clinical trial conducted in the Chinese PLA General Hospital (NCT04976218), CAR‐T cells that targeted EGFR were edited by CRISPR‐Cas9 to knock out transforming growth factor beta (TGF‐β), which is considered a major regulatory factor in the immunosuppressive tumor microenvironment (TME), and CAR‐EGFR‐TGFβR‐KO T cells may relieve the hostile TME in solids and improve the treatment effect. Antigen loss is the main reason for the failure of tumor immunotherapy; one of the strategies is to target more than one target simultaneously. A clinical trial conducted by the Chinese PLA General Hospital evaluated the feasibility and safety of universal bispecific CD19^+^CD20^+^ and CD19^+^CD22^+^ CAR‐T cells for treating relapsed or refractory B‐cell leukemia and lymphoma (NCT03398967).^152^

The well‐known immune checkpoints PD‐1 and CTLA‐4 can significantly inhibit the activity of T cells. Tumor cells can easily escape the immune response by virtue of this mechanism, resulting in poor therapeutic effects. Using gene‐editing tools to destroy endogenous immune checkpoint genes to enhance the antitumor effect is a novel treatment strategy that is widely used in the treatment of solid tumors. In 2016, the first clinical trial of PD‐1 knockout T cells for treating NSCLC was launched at West China Hospital of Sichuan University (NCT02793856). Investigators took advantage of CRISPR‐Cas9 to edit the PD‐1 gene of T cells ex vivo. After culturing and expanding in vitro, the T cells were reinfused into the subject, which supported the safety and feasibility of this therapy in NSCLC for the first time.^153^ Additionally, in a CRISPR‐related clinical trial led by Professor Carl June, researchers eliminated the genes encoding PD‐1 and endogenous TCR through CRISPR‐Cas9, effectively enhancing the effect of CAR‐T cells targeting melanoma (NCT03399448). At present, PD‐1 knockout T cells have also been used for treating advanced hepatocellular carcinoma (NCT04417764), invasive bladder cancer (NCT02863913), metastatic renal cell carcinoma (NCT02867332), esophageal cancer (NCT03081715), and EBV‐associated malignancies (NCT03044743), elevating the progress in cancer immunotherapy.^154^, ^155^, ^156^, ^157^

In addition to adoptive cell transfer therapy, many novel therapeutic strategies have been tested for treating tumors in preclinical studies. For example, the success of the SARS‐CoV‐2 mRNA vaccine depended on the advanced lipid nanoparticle (LNP) delivery system.^158^, ^159^ This efficient delivery vehicle has the potential to perform therapeutic gene editing in vivo. In a preclinical study, novel amino‐ionizable LNPs encapsulating Cas9 mRNA and gRNAs were injected into orthotopic glioblastoma to disrupt the polo‐like kinase 1 gene. In animal models, this strategy had positive and safe therapeutic outcomes.^160^ Gao et al. constructed a new Cas13a expression vector containing a nuclear factor κB (NF‐κB)‐specific promoter and U6 promoter. The expression of Cas13a is controlled by NF‐κB, which is widely overactivated in various cancers. Once the promoter is activated, the expression of endogenous oncogenes can be regulated by designing different sgRNAs within the vector.^161^ Furthermore, prime editors and some Cas variants circumvent specific adverse effects in traditional genome editing and catalyze the editing process without the requirement for DSBs.^162^

### 4.2 Infectious diseases caused by viruses

Genome editing is expected to become a powerful tool in antiviral therapy that acts by modifying infection‐related genes required for viral invasion and replication in host cells. Through gene editing, virus‐resistant immune or stem/progenitor cells can be produced, which could prevent or alleviate viral diseases.^163^

C‐C chemokine receptor type 5 (CCR5) and C‐X‐C chemokine receptor type 4 (CXCR4), the main auxiliary receptors for human immunodeficiency virus (HIV), play major roles in the initial infection and the establishment of stable infection, respectively. Additionally, homozygous carriers of the CCR5 Delta 32 mutation are naturally resistant to HIV infection, suggesting that the artificial deletion of CCR5 may be used to endow T cells with the feature of HIV infection resistance.^164^ Previous studies have used different genome editing tools to inactivate the CCR5 gene and CXCR4 gene on CD4+ T cells and CD34^+^ hematopoietic stem and progenitor cells (HSPCs) successfully,^165^, ^166^, ^167^ and these findings showed that these cells are not susceptible to HIV.^168^, ^169^ Among anti‐HIV therapies, the strategy of deleting the CCR5 gene through ZFN is relatively mature, and many clinical trials have been approved and undertaken. The first clinical trial using gene editing to treat HIV was led by Carl June in 2009. Researchers used ZFNs (SB‐728) to inactivate the CCR5 gene of autologous CD4+ T cells and reinfused these genetically modified T cells (SB‐728‐T) into 12 recruited patients. The results showed that except for one patient who exhibited serious adverse transfusion reactions, the remaining patients were tolerant to genetically engineered T cells,^170^ indicating that CCR5‐modified autologous T‐cell infusion is safe and feasible (NCT00842634). Subsequently, a plurality of clinical trials focused on determining the therapeutic dose and impact of SB‐728‐T to establish an efficacious clinical protocol (NCT03666871, NCT01044654, and NCT01252641). With the continued emphasis on the safety and effectiveness of immunotherapy, novel treatment strategies have emerged, including electrotransfecting CCR5‐specific ZFN mRNA to edit T cells (NCT02225665, NCT02388594) or knocking out the CCR5 gene in CAR‐T cells to treat HIV (NCT03617198). Scientists wished to explore the effects of genetically modified T cells on HIV infection resistance under these strategies. In addition to genetically modifying T cells, scientists would like to modify hematopoietic stem or progenitor cells to create a cell pool in vivo that can continue to produce T cells resistant to HIV infection. In a clinical study, researchers from the City of Hope Medical Center delivered CCR5‐specific ZFN mRNA into HSPCs to evaluate its safety in patients infected with HIV‐1 (NCT02500849).^171^ Moreover, in a recent clinical study, Chinese scientists designed a stable CRISPR‐Cas9 system to edit the CCR5 gene of donor‐derived CD34^+^ hematopoietic stem cells (HSCs) and infused these cells back into the patient.^102^ They then evaluated the feasibility and safety of this therapeutic strategy in patients infected with HIV. After patients with ALL and HIV infections received the infusion of these autologous CCR5‐deficient HSPCs, the acute lymphocytic leukemia was in complete remission, and CCR5‐deficient donor cells remained in the body for more than 19 months. This case indicated that in humans, CCR5‐inactivated HSPCs contributed to long‐term hematopoietic system reconstruction.^172^ However, the low efficiency of gene editing limits the therapeutic effect, suggesting that the focus of this research may be to improve the editing efficiency. The above cases have supported the immense potential of gene‐editing technology in treating HIV and put forward further requirements for the efficiency of gene editing and the optimization of the treatment scheme.

Another strategy for antiviral therapy based on gene manipulation is to target the viral genome associated with viral replication and assembly directly. In addition to addressing HIV infection, gene‐editing platforms have been applied to other causative agents, including HPV, hepatitis B virus (HBV), and EBV. The main etiological factors of cervical cancer are some specific oncoproteins. The HPV*E6/E7* genes encode important oncoproteins related to the neoplastic transformation of the disease. Multiple clinical trials have attempted to introduce different gene‐editing modules to target and destroy the DNA of HPV16/18 E6 or E7 directly in vivo. The researchers hope that the tumorigenic process will be reversed in situ and that the incidence of cervical cancer will be reduced (NCT03226470, NCT03057912, and NCT02800369).^173^, ^174^, ^175^ A similar idea was adopted for the elimination of HBV. To inhibit HBV replication, researchers used lentivirus to deliver CRISPR‐Cas9 nuclease and HBV‐specific sgRNA into HepG2 cells bound to HBV.^176^

The COVID‐19 led the World Health Organization to declare a pandemic in March 2020, and it remains a significant threat to human health worldwide. Zhang et al. designed a novel diagnostic tool termed SHERLOCK to detect the nucleic acids of viruses.^126^ Inspired by the SHERLOCK system, American scientists transformed Cas13a into an antiviral agent that was programmed to detect and destroy RNA viruses in human cells in another novel study. The antiviral activity of Cas13a and its diagnostic ability were combined to construct a system that may be used to diagnose and treat viral infections, which is termed Carver (i.e., Cas13‐assisted restriction of viral expression and readout).^177^

### 4.3 Hematological diseases

Many hematological disorders are caused by genetic mutations, including thalassemia, hemophilia, and SCD. The correction of erroneous gene mutations can be accomplished by gene‐editing technology, which undoubtedly holds promise for treating hereditary hematological diseases.

The *HBB* gene mutation on chromosome 11 reduces β globin chain production, which in turn causes β‐thalassemia.^178^ HSCs have significant advantages in reconstructing or restoring human hematopoietic function. A clinical experiment attempted to modify induced HSCs from patients to correct the mutated *HBB* gene and then transfuse them back into the patients to restore their normal hemoglobin production ability (NCT03728322). The presence of fetal hemoglobin (HbF) can in some cases alleviate the symptoms of thalassemia, but HbF production is suppressed by BCL11A.^179^, ^180^ Thus, knocking this gene out or down leads to a suitable approach to treating SCD and beta‐thalassemia. An experimental CRISPR‐based therapy (known as CTX001) invented by CRISPR Therapeutics has undergone two clinical trials for treating SCD and thalassemia (NCT03655678; NCT03745287). The key strategy underlying CTX001 is to use CRISPR‐Cas9 to modify the BCL11A gene within CD34^+^ human hematopoietic stem and progenitor cells to increase the production of HbF in vivo. Sangamo Therapeutics and Sanofi also conducted two clinical trials using a similar strategy. However, ZFNs were used instead of a CRISPR/Cas system for genetic modification (NCT03432364, NCT03653247). Furthermore, the appearance of base editors that have the capacity to manipulate a single base without DSBs provides an attractive option to cure sickle‐cell disease. Liu et al. generated a bespoke base editor to convert the pathogenic gene (*HBB* ^*S*^, hemoglobin subunit beta allele) into a nonpathogenic gene (*HBB* ^*G*^, Makassar β‐globin). Based on this strategy, approximately 80% of hematopoietic stem and progenitor cells derived from patients could be engineered in vitro. Furthermore, humanized SCD mice that received edited HSPCs exhibited reduced splenic and hematologic pathology, compared to untreated mice.^120^ Hemophilia B is a blood clotting disorder caused by mutations in the clotting factor XI gene.^181^ In a Phase I clinical trial, researchers introduced ZFNs to patients intravenously. The editing agents could install the correct clotting factor XI gene into the albumin locus of hepatocytes. The aim of the clinical trial is to produce a permanent secretion of coagulation factor XI in the body (NCT02695160).

### 4.4 Metabolic disorders

Mucopolysaccharidosis is a metabolic disorder caused by the congenital absence of lysosomal enzymes. Furthermore, most mucopolysaccharidoses are autosomal recessive. This disease has seven typical clinical types, and two clinical trials for MPS I and MPS II are currently underway.^182^ MPS I is primarily due to a lack of α‐L‐iduronidase, while the shortage of iduronate sulfatase primarily causes MPS II. Angamo Therapeutics Inc. used AAV‐derived vectors to deliver gene‐editing components into hepatocytes. These ZFNs could insert a normal α‐L‐iduronidase gene or iduronate 2‐sulfatase gene into albumin sites to obtain lifetime lysosomal enzyme production capacity (NCT02702115, NCT03041324). Additionally, several published reports have demonstrated the safety and effectiveness of the CRISPR system in treating MPS I.^183^

ADA deficiency is a metabolic disease caused by mutations in the *ADA* gene.^184^, ^185^ In a previous clinical study, the patient‐derived HSPC genome was corrected by introducing ADA complementary DNA, and the corrected cells were then autologously transplanted into patients (NCT01380990). It is worth noting that the gene fragment was introduced by lentivirus, which may carry a risk of insertion mutation.

In Type 1 diabetes mellitus, the islet cells that produce insulin in the pancreas are mistakenly attacked by effector T cells, failing to respond to blood glucose changes. Additionally, regulatory T (Treg) cells cannot properly interfere with this false attack, which ultimately leads to the progression of diabetes. Researchers use gene‐editing technology to target the forkhead box P3 (*FOXP3*) gene in human T cells, inserting a robust enhancer/promoter proximal to the first coding exon. The persistent activation of the *FOXP3* gene allows T cells to be artificially intervened to transform into Treg cells. These edited Treg cells have the potential to stop the negative reaction within the pancreas for treating diabetes. The clinical benefits of gene‐editing therapeutics for Type 1 diabetes still require more study.^186^

### 4.5 Neurodegenerative disorders

NDs are caused by the loss of neuronal structure or function in the brain and spinal cord, including AD, PD, HD, and amyotrophic lateral sclerosis (ALS). The number of patients gradually increases due to the lack of effective early diagnosis and successful therapeutic intervention. Additionally, as limited by the understanding of the pathogenesis of NDs, the exploitation of therapeutics is extremely challenging. Genetic mutation and the aggregation of misfolded proteins are considered potential pathogenic mechanisms of ND.^187^, ^188^ Therefore, correcting gene or protein errors based on the gene‐editing platform is promising for exploring and treating NDs. There are thousands of clinical trials on NDs. In addition to a few approved monoclonal antibodies, no ND treatments have displayed promising clinical outcomes. In addition, many results of clinical trials have yet to be verified.^189^ Currently, gene‐editing therapeutics for NDs are still in their infancy. Thus, we primarily present some preclinical research progress here.

AD is one of the most common chronic NDs, and its primary pathological features are amyloid plaques and neurofibrillary tangles, which eventually lead to a severe cognitive disorder.^190^ Swedish scientists reported that they used the CRISPR‐Cas9 system to correct the mutation of the gene encoding APP in a patient's cells. This change could lead to a treatment for AD.^191^ In addition, scientists have generated several antibodies targeting β‐amyloid protein for active or passive immunity. As significant components, apolipoprotein E and PSEN participate in the disease process of AD and can serve as potential targets for the gene‐editing system.^192^ HD is a rare ND with an autosomal dominant inheritance caused by an abnormality in the *HTT* gene.^193^ The accumulation of abnormal metabolites (the huntingtin protein) damages the cerebral cortex, leading to mental decline and the loss of athletic ability.^194^ Although tetrabenazine is approved for treating HD, the effect of the drug is limited. Current treatment strategies to alleviate HD primarily include directly replacing damaged or lost neurons, knocking out or silencing genes that express abnormal proteins, and reducing the harm caused by the huntingtin protein to protect the surviving neurons.^195^, ^196^ The main pathological feature of PD involves the loss of dopaminergic neurons in the substantia nigra pars compacta and brain cell death.^197^, ^198^ As there is no cure for PD, the current conventional treatment strategy is to compensate for the loss of dopamine artificially; however, this approach only relieves the symptoms. A recent study used the CRISPR‐Cas9 system to inactivate the alpha synuclein (*SN* *CA*) gene encoding alpha‐synuclein, which is related to the formation of Lewy bodies. Compared to unedited cells, edited stem cells can differentiate into dopamine‐producing neurons and do not produce Lewy bodies when subjected to specific chemical stimuli. This work reaffirms the potential of gene editing‐mediated cell replacement therapy in treating NDs.^199^, ^200^ ALS is characterized by the extensive degeneration of motor neurons in the spinal cord, brain stem, and cerebral cortex.^201^, ^202^ The current treatment strategy replaces damaged motor neurons with neural stem cells that secrete neurotrophic factors. Mutations in the gene encoding superoxide dismutase 1 are one of the pathological causes of ALS.^203^ Researchers from the University of California, Berkeley, verified that the deletion of the superoxide dismutase type 1 (*SOD1*) gene could improve the retention of motor neurons in a rat model.^204^, ^205^

To conclude, the lack of feasible treatment options for NDs is temporary. Gene‐editing platforms allow us to understand the occurrence of diseases at the genetic level and to establish more complete disease models.

### 4.6 Ocular disorder

The great potential of using limbal stem cells for treating corneal injury is described above. Gene therapy has also made significant progress in treating hereditary eye diseases. LCA is a major hereditary disease that causes blindness in children, of which LCA10 is the most common and the most serious type. In 2017, the US FDA approved a gene therapy called Luxturna (voretigene neparvovec‐rzyl) to treat patients with LCA2 with *RPE65* gene mutations.^206^, ^207^, ^208^ The pathological cause of LCA10 is a mutation of the *CEP290* gene (the most common mutation is termed p.Cys998X).^113^ In 2020, Allergan and Editas Medicine Inc. initiated a clinical trial to treat LCA10 using AGN‐151587 (EDIT‐101), which could eliminate the mutation in *CEP290* using Cas9 effectors, combined with the AAV vector (NCT03872479).

## 5 THERAPEUTIC GENOME EDITING: OPPORTUNITIES AND CHALLENGES COEXIST

### 5.1 Genome‐editing technologies increase the potential to treat genetic diseases

According to the latest data from the OMIM Gene Map Statistics, more than 4000 genes will cause morbid phenotypes in humans.^209^ A total of millions of people worldwide suffer from genetic disorders. Unfortunately, most of them are unable to receive optimal treatments because of the dearth of therapeutic drugs and diagnostic methods.^210^ Traditional drug development to treat rare genetic diseases costs vast amounts of money and time, forcing many pharmaceutical companies to retreat. Exploiting a novel treatment for genetic diseases is urgently needed.^211^, ^212^ The plight of unmet medical needs is currently expected to be addressed by therapeutic genome editing. Emerging gene‐editing technologies have expanded our abilities to manipulate the gene sequences of eukaryotic cells.^30^, ^36^, ^58^, ^60^, ^175^ These molecular tools make it possible to cure genetic diseases by correcting or removing the errors within genome sequences.

Notably, therapeutic products based on gene‐editing technologies have offered unique advantages over traditional small molecular drugs and antibodies.^147^ A prominent strength of gene therapy is that it can offer a customized therapeutic schedule for individuals who have limited or no treatment options.^212^ Of course, personalized clinical procedures may be restricted by regulatory guidelines and have a heavy financial burden. The promising clinical benefits, however, will encourage regulatory authorities to adjust their policies toward these emerging therapies.^213^ The treatment strategies for most gene‐editing therapies can be briefly summarized in several steps, including screening, synthesis, delivery, the detection of target gene sequences and industrialized production.^214^ Compared to small‐molecule targeted drugs and monoclonal antibodies, nucleic acid sequences are easy to design and less expensive.^215^ Additionally, the expanding gene‐editing arsenal has provided many versatile tools to manipulate the gene sequence as needed.^216^ Last, the desired gene cargoes and editing tools could be transferred into cells efficiently using various vehicles, including viral or nonviral vectors.^217^ Simplified research and development processes can reduce time and vastly accelerate the progress of gene therapy.^218^

### 5.2 Several challenges limit the development of clinical applications

#### 5.2.1 Efficiency

**FIGURE 4:**
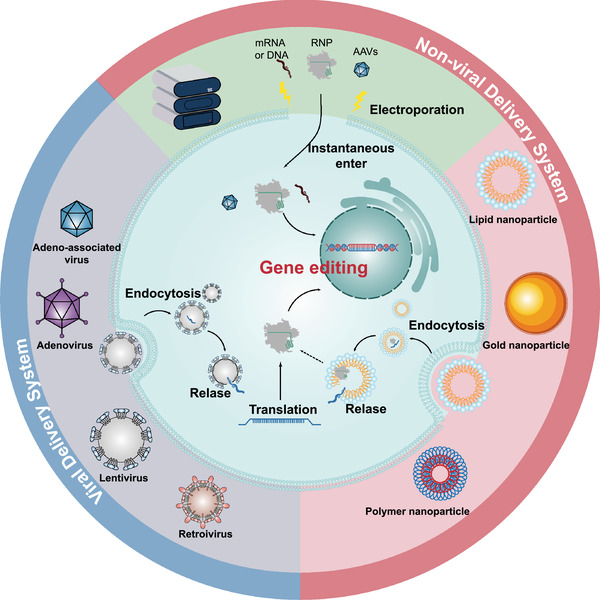
Different delivery systems for therapeutic gene‐editing machinery. The current delivery strategies can be classified into two types: virus‐based and nonviral delivery systems. Different viral vectors, including those of adeno‐associated virus (AAV), adenovirus, lentivirus, and retrovirus, have been leveraged to deliver gene‐editing components and template DNA. The nonviral delivery system can be subdivided into two strategies: electroporation and nanoparticle injection

In addition to safety, the wide applications of genome editing depend on its efficiency. The common factors affecting editing efficiency include the following: (i) the target cell type and cellular environment, (ii) the optimal choice of gene‐editing tools, (iii) the competition of different cellular DNA repair pathways, and (iv) the technologies for delivering editing components.^219^, ^220^, ^221^, ^222^ Of these factors, the efficient delivery of genome editing machinery in vitro and in vivo is the foundation of successful gene‐editing therapeutics.^217^ Therefore, we will focus on discussing the development of delivery strategies. Previous studies have developed multiple delivery methods to transfer macromolecules (such as proteins, DNA, siRNA, or mRNA) into the cell.^145^, ^223^ The current delivery strategies can be classified into two formats: virus‐based delivery systems and nonviral delivery systems (Figure 4).^145^

A variety of viral vehicles, including AAVs, lentiviruses, adenoviruses, and retroviruses, have been applied to deliver gene‐editing components into target cells of interest.^144^, ^224^, ^225^, ^226^ Most currently, AAVs and lentiviruses, depending on some unique advantages, have been widely used in clinical trials.^227^, ^228^, ^229^ Natural AAV vectors have 11 serotypes, which possess inherent tropisms for different tissues.^227^ Because of the tropism for the eye, AAV2 has been approved by the FDA for treating degenerative retinal disorders.^230^, ^231^ Moreover, AAV vectors could reduce the risk of genomic mutation because the gene cargos carried by AAV cannot integrate into the host genome. Although many attractive features of AAVs have been noted, some existing challenges are still awaiting address. The first challenge is the limited packing capacity of AAV vehicles.^232^ Most AAV vectors can encode up to 4.4 kb of exogenous DNA, less than other tools.^233^ Sometimes, the carrying capacity can accommodate only one CRISPR‐Cas endonuclease gene without other space for sgRNA or donor templates. A feasible solution is using two vectors to deliver the whole system with the potential to reduce efficiency.^234^, ^235^ The employment of smaller engineered Cas variants is an alternative strategy to tackle the capacity limit and improve efficiency.^236^ Second, the preexisting adaptive immunity to natural serotypes hinders the in vivo delivery effectiveness of AAV vectors.^237^ However, capsid engineering may circumvent this limitation.^238^, ^239^ In addition, the transgene delivered by AAVs has long‐term expression in target cells, increasing the editing efficiency but simultaneously posing a potential risk of off‐target toxicity or immunotoxicity.^240^

Lentivirus, the replication‐incompetent vector with a 10 kb packing capacity,^241^ is capable of introducing therapeutic DNA into primary cells, such as T cells or HSCs.^242^ For instance, in four approved CAR‐T therapies, CAR genes were introduced into lymphocytes using lentivirus.^243^, ^244^, ^245^, ^246^ However, the random integration of exogenous genes has restricted the usage range of lentivirus.^247^ Moreover, some characteristics, including the narrow scope of the target specificity and poor delivery efficiency, are also the current barrier to lentivirus applications. At present, the appearance of novel lentiviral vectors, such as integrase‐defective lentiviral vectors, perhaps provides the potential to address these issues.^248^, ^249^, ^250^ In general, for most virus‐based delivery systems, the existing manufacturing technique cannot adapt to the changing requirements.^251^ Technological innovation is critical to improving the efficiency of viral vectors. For example, the potency of AAVs has been enhanced by protein engineering.^252^

Nanoparticles have been found to be suitable for delivering genome editors and provide many options for delivery vehicles, including polymer, lipid, and gold nanoparticles.^253^, ^254^, ^255^ Unlike viral vectors that only accommodate cDNA encoding therapeutic effectors, nonviral delivery systems possess a tunable carrying capacity for various transportation objects.^256^ For instance, Cas9 RNPs encapsulated in cationic LNPs are engaged by cells via endocytosis and micropinocytosis.^257^ A feasible approach led to moderate editing outcomes in a mouse model.^258^ Although the editing efficiency of lipid‐mediated nanoparticles is not equivalent to that of virus vectors, nanoparticles present many unique advantages. In addition to their low cost and convenient assembly, one major strength is their transient expression in target cells because nanoparticles cannot induce a long‐term reaction, reducing the risk of off‐target effects or immunotoxicity. However, the injected nanoparticles primarily accumulate in the liver or spleen, causing undesired toxicities, which limits their clinical application. Gold nanoparticles are considered an attractive candidate for tissue‐targeted delivery because of their modifiable surface characteristics.^259^, ^260^ An alternative strategy to reduce toxicity is electroporation.^261^, ^262^ This microbiological technology increases the permeability of the cell membrane by using a current pulse, allowing gene‐editing components to enter cells. The straightforward delivery procedure improves the editing efficiency in cells while preserving cell viability as much as possible.^263^ However, the scenarios for electroporation use are limited, and in vivo delivery is impractical. Some investigators have tried to combine different delivery methods to improve editing efficiency.^264^ Dai et al. established a system to generate CAR‐T cells with immune checkpoint gene knockout using electroporation and AAV6 vectors. These attempts will accelerate the development of delivery systems.^265^ In summary, even though current delivery systems have some disadvantages, emerging delivery strategies will offer more options for us to achieve ideal gene editing.

#### 5.2.2 Safety considerations

However, as previously mentioned, an increasing number of clinical applications and tests have demonstrated the availability of gene editing.^153^, ^266^, ^267^, ^268^ There are still several technical limitations that hinder the broad clinical utility of genome editing. Scientists have made a great deal of effort to break through these limitations by developing various hybrid strategies.

The safety of therapeutic gene editing is a key concern for investigators and patients.^147^, ^269^, ^270^ The primary security risks arise from the discrepant editing accuracy and immunogenicity of gene‐editing effectors or delivery reagents.^271^ Ideal editing outcomes involve the precise installation of desired mutations within the target site without creating any byproducts. However, due to deficient target specificity, DNA cleavage may occur at the wrong genome locations that have similar features to the intended editing sequence. In addition, the DSBs generated by various gene‐editing nucleases generally induce different modes of DNA repair, including NHEJ and/or HDR.^272^ In some cases, even if the desired DSBs appear in the target site, the unpredictable and complex repair process may also cause undesired mutagenesis.^221^, ^273^ For instance, once off‐target events occur in cardiac cells, even the frequency is small, which will cause irreversible problems.^274^ To improve the accuracy and precision of gene editing, some researchers have tried to generate new orthologs with greater specificity,^275^ such as SpCas9 and xCas9.^25^, ^276^, ^277^, ^278^ Another approach is to avoid producing DSBs.^70^, ^279^ Liu et al. demonstrated that base editors and prime editors could install a single base mutation or small fragment within genome sequences without reliance on DSBs and HDR.^58^, ^70^ Moreover, it is worthwhile to establish effective methods for monitoring off‐target events in humans.^280^ Some current evidence has suggested that in vitro analysis of off‐target editing in primary cells can serve as a guide for in vivo situations.^280^

The immunogenic toxicity of editing proteins is frequently mentioned in association with CRISPR‐Cas systems. The engineered Cas nucleases were remolded based on natural Cas proteins derived from some bacteria.^19^ If individuals have ever been infected by these pathogens, the engineered editing effectors have the potential to be captured by preexisting antibodies and trigger inflammation and other unknown side effects.^281^, ^282^ Although some studies have detected specific antibodies and preexisting adaptive immunity to Cas9 in humans,^282^, ^283^ we still need sufficient evidence to clarify the occurrence mechanism of immunotoxicity caused by preexisting antibodies.^284^ Together, these studies are helping to identify more secure proteins for gene editing in the clinic. The immune response to the delivery modality has been discussed in the previous section.

#### 5.2.3 Ethical challenges

Many studies have highlighted the remarkable therapeutic benefits and promising future of gene‐editing therapy. However, we cannot focus only on short‐term successes and ignore the unique ethical challenges.^285^ Although germline changes have been completed in plants or some animals,^286^, ^287^ there is some controversy over human germline alterations. There is no doubt that human germline genome editing must be rigorously regulated.^288^ In the absence of sufficient understanding, all scientists should stand in awe of human germline genome editing. Of course, some rational attempts should be allowed under strict supervision.

## 6 FUTURE OUTLOOK AND CONCLUSION

A profound revolution and innovation have been driven by gene‐editing technologies in many fields, including agriculture, medicine, biotechnology, and the manufacturing industry. In the last decade, more efficient and versatile gene‐editing platforms have been established and now offer us a powerful tool for investigation and genome engineering in eukaryotic cells. As a research tool, various gene‐editing agents have enabled us to characterize and understand the functions of normal genes. In addition, scientists have the opportunity to screen disease‐causing mutations and elucidate the pathogenesis of some rare genetic diseases. As a diagnostic tool, CRISPR‐Cas systems have been re‐engineered to detect viral nucleic acids, such as SARS‐CoV‐2. The successful application of gene editing in the diagnosis of infectious diseases indicates that CRISPR‐Cas nucleases have the potential to be developed into an accurate and rapid diagnostic tool for other diseases. The most inspiring application of gene editing is in the field of gene or cell therapy. The correction and alteration of disease‐causing gene mutations offer the possibility of treatment or even a permanent cure for some genetic disorders. Emerging gene manipulation tools have addressed many technological issues associated with immunotherapy. For instance, some immunotherapies, particularly CAR‐T therapy, are poised to create a “paradigm shift” in malignant tumours. To date, we have witnessed many promising clinical outcomes and have accumulated increasing clinical experience.

Despite the fact that gene‐editing therapeutics have been subject to tremendous progress in clinical applications, several formidable problems need to be approached before the ultimate aspiration of curing all genetic disorders can be fulfilled. First, scientists continue to increase the accuracy and efficiency of existing gene‐editing agents in parallel with innovations and developments of novel technologies. Additionally, we still emphasize the urgent need for optimal delivery approaches, which are the major barriers to achieving efficient gene manipulation in vivo. Furthermore, bioethicists have stressed that the original intention of genome editing is to correct morbific errors rather than eliminate differences. Therefore, we should be vigilant about the deliberate or unintentional misuse of these customized tools.

Scientific technology offers a profound opportunity to reshape medical treatments. Realizing the full potential of gene‐editing technology depends not only on the efforts of scientists and clinicians but also on the support of the government and other stakeholders. It is foreseeable that gene‐editing technology will provide a novel avenue for health care in the future.

## CONFLICT OF INTEREST

The authors declare that there are no conflicts of interest.

## ETHICS STATEMENT

The authors declare that human ethics approval was not needed for this study.

## AUTHOR CONTRIBUTIONS

W.W. conceived and presented the article idea and supervised the whole work. W.Z. collected the data and wrote the first draft of the manuscript. J.Y., Y.Z., and X.H. participated in editing the manuscript. W.W. provided important suggestions for manuscript writing. All authors participated in the work and approved the manuscript for publication.

## Data Availability

The data included in this study are available upon request from the corresponding author.
